# Pilot Study on Folate Bioavailability from a Camembert Cheese Reveals Contradictory Findings to Recent Results from a Human Short-term Study

**DOI:** 10.3389/fnut.2016.00009

**Published:** 2016-04-08

**Authors:** Sabine Mönch, Michael Netzel, Gabriele Netzel, Undine Ott, Thomas Frank, Michael Rychlik

**Affiliations:** ^1^Lehrstuhl für Lebensmittelchemie, Technische Universität München, Freising, Germany; ^2^Department of Human Nutrition, Institute of Nutrition, Friedrich Schiller Universität Jena, Jena, Germany; ^3^Kuratorium für Dialyse und Nierentransplantation e.V., Jena, Germany; ^4^Private Consultant, Bad Soden, Germany; ^5^Chair of Analytical Food Chemistry, Technische Universität München, Freising, Germany

**Keywords:** bioavailability, Camembert cheese, humans, folates, stable isotope dilution assay

## Abstract

Different dietary sources of folate have differing bioavailabilities, which may affect their nutritional “value.” In order to examine if these differences also occur within the same food products, a short-term human pilot study was undertaken as a follow-up study to a previously published human trial to evaluate the relative native folate bioavailabilities from low-fat Camembert cheese compared to pteroylmonoglutamic acid as the reference dose. Two healthy human subjects received the test foods in a randomized cross-over design separated by a 14-day equilibrium phase. Folate body pools were saturated with a pteroylmonoglutamic acid supplement before the first testing and between the testings. Folates in test foods and blood plasma were analyzed by stable isotope dilution assays. The biokinetic parameters *C*_max_, *t*_max_, and area under the curve (AUC) were determined in plasma within the interval of 0–12 h. When comparing the ratio estimates of AUC and *C*_max_ for the different Camembert cheeses, a higher bioavailability was found for the low-fat Camembert assessed in the present study (≥64%) compared to a different brand in our previous investigation (8.8%). It is suggested that these differences may arise from the different folate distribution in the soft dough and firm rind as well as differing individual folate vitamer proportions. The results clearly underline the importance of the food matrix, even within the same type of food product, in terms of folate bioavailability. Moreover, our findings add to the increasing number of studies questioning the general assumption of 50% bioavailability as the rationale behind the definition of folate equivalents. However, more research is needed to better understand the interactions between individual folate vitamers and other food components and the potential impact on folate bioavailability and metabolism.

## Introduction

Folates are “key players” in the one-carbon metabolism pathways and thus are involved in DNA synthesis, amino acid metabolism, and methylations (1). However, mean intake of folate in many countries is considered to be below the dietary recommendations for humans. Low dietary intake of folate is linked with the risk of neural tube defects (2) and is suspected to be associated with the development of certain forms of cancer (3), Alzheimer’s disease (4), and cardiovascular disease (5). Obeid and colleagues concluded in a recently published study that there is an epidemic of folic acid-preventable spina bifida and anencephaly in Europe and that mandatory fortification would improve public health in Germany and other European countries (6). Therefore, many countries in the European Union favor the consumption of foods endogenously high in folates or increasing endogenous folate content in foods. However, apart from folate content alone, bioavailability appears to be the challenge if folate supply from foods is intended to be increased. The current dietary recommendations are based on the findings of Sauberlich et al. (7), who determined in a long-term human study a 50% bioavailability of native folates from foods relative to pteroylmonoglutamic acid. This value has since been questioned as subsequent studies revealed bioavailabilities of folates, e.g., from spinach of 89–113% (8), from citrus fruits and vegetables of 98% (9) relative to pteroylmonoglutamic acid, or from broccoli and strawberries of 99–120% relative to 5-CH_3_-H_4_folate (10). This prompted us to conduct a recently published human study on folate bioavailability by using stable isotope dilution assays for analysis of plasma folate and an area under the curve (AUC) approach (11). A surprisingly high inter-individual variation in folate bioavailability and also highly different bioavailabilities of the test foods ranging from 8.8% for Camembert cheese to 73% for spinach relative to pteroylmonoglutamic acid could be observed (12). However, we did not investigate the potential variation in folate bioavailability within the same test food, e.g., Camembert cheese. Therefore, the aim of the present pilot study was to compare the oral bioavailability and biokinetic data between pure compound of pteroylmonoglutamic acid as the reference and native folate from a low-fat type Camembert cheese of a brand different to that used in the aforementioned human study (12). The bioavailability data of the cheese, which is a popular and significant dietary source of folate in Germany (13), were obtained from analyses of 5-CH_3_-H_4_folate in plasma following oral administration.

## Materials and Methods

### Chemicals

The following chemicals were obtained commercially from the sources given in parentheses: rat serum (Biozol, Eching, Germany), chicken pancreas (Difco, Sparks, MD, USA), acetic acid, acetonitrile, sodium phosphate dibasic dihydrate, formic acid, hexane, methanol, potassium phosphate monobasic, sodium hydroxide, sodium chloride (Merck, Darmstadt, Germany), alpha-amylase, ammonium formate, ascorbic acid, pteroylmonoglutamic acid, 4-morpholineethanesulfonic acid (MES), 2-mercaptoethanol, protease type XIV, sodium acetate (Sigma, Deisenhofen, Germany), (6*S*)-tetrahydrofolic acid, calcium (6*S*)-5-methyltetrahydrofolate, 10-formylfolic acid, and (6*S*)-5-formyltetrahydrofolic acid (Schircks, Jona, Switzerland). All chemicals were at least of analytical-reagent grade. [^2^H_4_]-5-methyltetrahydrofolic acid, [^2^H_4_]-5-formyltetrahydrofolic acid, [^2^H_4_]-tetrahydrofolic acid, [^2^H_4_]-10-formylfolic acid, and [^2^H_4_]-pteroylmonoglutamic acid were synthesized as previously reported (14).

### Test Foods

A low-fat Camembert cheese (16 g fat/100 g) was purchased at a local supermarket in the city of Munich, Germany. The pteroylmonoglutamic acid solution was prepared by suspending pteroylmonoglutamic acid (2.0 mg) in tap water, which was then alkalized with dilute sodium hydroxide until all solids were dissolved and then adjusted to pH 7 with dilute hydrochloric acid followed by adjustment to volume (1 L) with tap water. The Camembert cheese under study was assessed in two different ways: (a) whole cheese samples were frozen in liquid nitrogen and homogenized in entirety and (b) cheese samples were segmented into rind and dough and analyzed separately by stable isotope dilution assays as previously described (15). Quality control was performed by assessing recovery, precision, linearity, LOD, limit of quantification (LOQ), and the analysis of dried, mixed vegetables as certified reference material (16).

### Plasma

Plasma samples were analyzed by stable isotope dilution assays using phenyl SPE cleanup as detailed by Mönch et al. (17). Briefly, aliquots of plasma (400 μL) were spiked with [^2^H_4_]-5-methyltetrahydrofolic acid (5 ng in MES buffer) and then overlaid with ammonium formate buffer (600 μL) and equilibrated for 30 min at room temperature and subjected to cleanup on phenyl SPE cartridges (Discovery DSC-ph, 100 mg, 1 mL, Varian, Darmstadt, Germany). Folates were eluted from SPE columns with 0.5 mL of a mixture of aqueous sodium chloride (5%) and sodium acetate (100 mmol/L; 1% ascorbic acid). The lower limit of quantification (LLOQ) was 0.37 nmol/L.

### Pilot Study and Ethical Permission

Two healthy, non-smoking Caucasian female subjects participated in the pilot study with a mean (±SD) age of 25.5 (±2.1) years and a mean (±SD) body mass index of 20.9 (±2.1) kg/m^2^. Ethical permission was obtained by the Ethics Committee of the Friedrich Schiller University Jena, Faculty of Medicine (code 1415-09/04). The study was carried out in accordance with the recommendations of the Ethics Committee given above with written informed consent from all subjects. All subjects gave written informed consent in accordance with the Declaration of Helsinki. To improve the uniformity among the two subjects and subsequently the precision of bioavailability estimates, a pteroylmonoglutamic acid supplement (800 μg/day = 1.8 μmol/day) was given for 14 days before the first testing and between the testings and was discontinued 2 days prior to the start of the study (12). The study had a randomized cross-over design, and each subject had the following experimental treatments separated by a 14-day equilibrium phase: 448-nmol total folates *via* Camembert cheese (200 g) and 453-nmol pteroylmonoglutamic acid *via* orally administered pteroylmonoglutamic acid solution (100 mL) serving as reference treatment. After an overnight fast, volunteers took the Camembert cheese or drank the pteroylmonoglutamic acid solution, respectively, together with one slice of toast bread. During the experimental treatment periods (24 h), the consumption of water was allowed *ad libitum*, and two further standardized and virtually folate-free study meals consisting of wheat bread (nine slices or 500 g), butter (100 g), and honey (250 g) were offered for lunch and dinner. Venous blood samples (9 mL) were drawn in EDTA-coated tubes (Sarstedt, Nuernbrecht, Germany) predose, as well as 1, 2, 3, 4, 5, 6, 8, 10, 12, and 24 h after the administration of Camembert cheese or reference solution. Blood was centrifuged (10 min/2000 × *g*/4°C) to separate plasma from red blood cells. Plasma was stored frozen at −24°C until further preparation and analysis.

### Biokinetic Calculations

**Figure 1:**
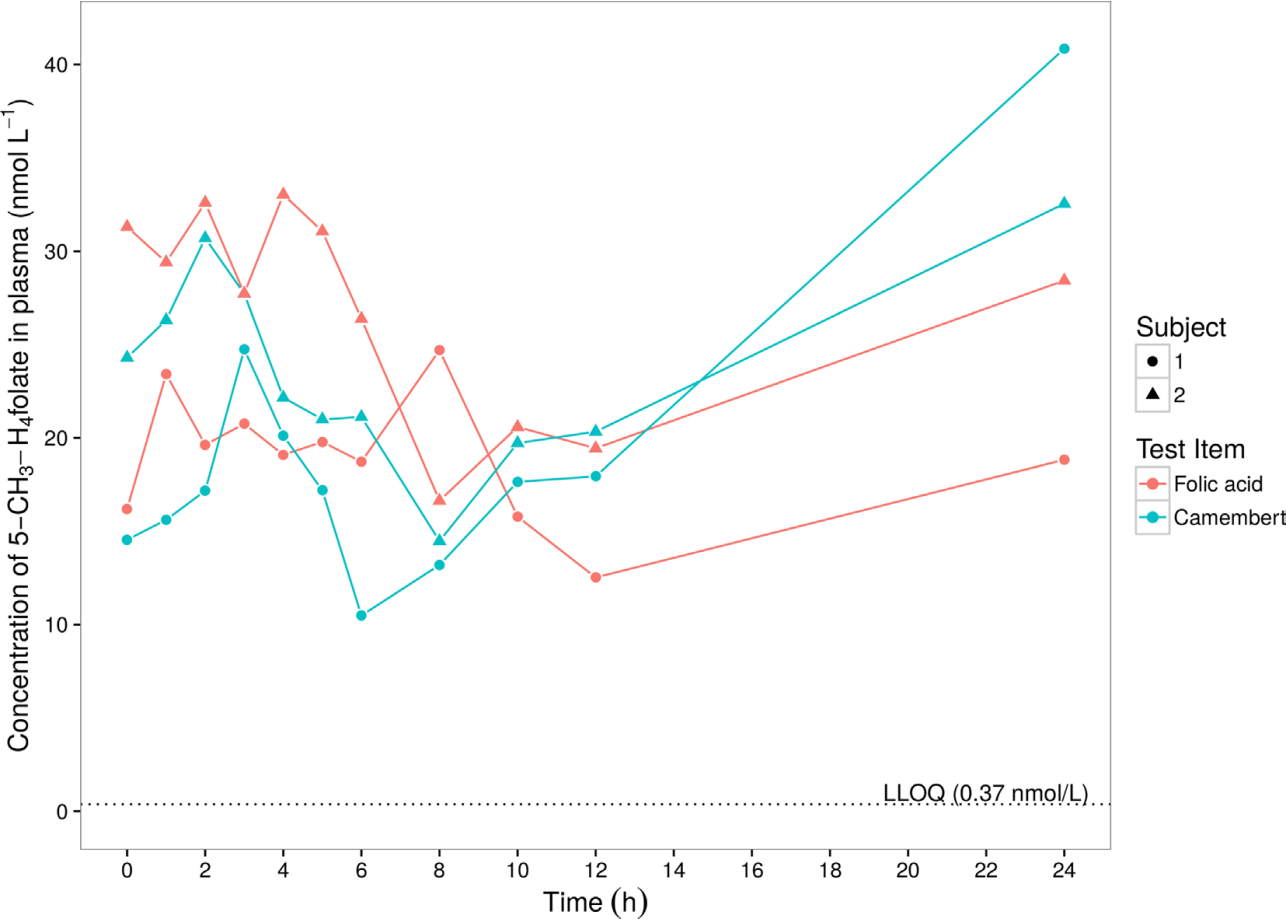
**5-Methyltetrahydrofolate levels in plasma of two volunteers after intake of folic acid solution or Camembert cheese**.

Plasma 5-CH_3_-H_4_folate was evaluated. In the first step, the individual predose (*t* = 0 h) 5-CH_3_-H_4_folate plasma concentrations were subtracted from all subsequent values. Negative concentrations that might result from this procedure were discarded. The predose-corrected concentrations were subjected to a standard non-compartmental analysis in order to derive *C*_max_ (observed maximum plasma concentration), *t*_max_ (time of *C*_max_), and AUC (area under the plasma concentration–time curve) (18). The calculation of the AUC was done by linear trapezoidal rule and was limited within the interval between 0 and 12 h after dose, because after 12 h, a somewhat unexpected and marked increase up to 24 h after dose occurred (see Figure 1). Concentrations below the LLOQ were treated as 0.

## Results

For determining the exact folate dose, we analyzed the Camembert cheese for individual folate vitamers and found considerably lower total folate content and a rather different folate profile compared to the Camembert cheese (different brand) assessed in our previous study (12). Whereas the latter contained as much as 647 nmol/100 g total folate and its major folate vitamer was tetrahydrofolate, the total folate content of the Camembert cheese in the present study was only 224 nmol/100 g, and its major vitamer was 5-formyltetrahydrofolate (Table 1). The time course of the 5-CH_3_-H_4_folate (non-predose-corrected) concentrations in plasma is shown in Figure 1. After intake of Camembert cheese, the concentrations increased up to 2–3 h after dose and decreased below the predose values to a local minimum at 6 and 8 h after dose. Thereafter, the concentrations increased continuously up to the next morning, i.e., 24 h after intake, well above the predose values. After intake of folic (pteroylmonoglutamic) acid solution, the curves showed a fluctuating increase with maxima reached within the region between 2 and 8 h after dose. At 8 and 12 h after dose, concentrations reached a minimum which was below the predose values in both subjects, followed by a continuous increase up to the next morning. Due to sufficient reducing and methylation capacity in intestinal epithelium and the liver, no other folate vitamers than 5-CH_3_-H_4_folate were detectable in circulating plasma. The increase in plasma folate concentration at late sampling times after folate dosage was also observed in our previous study (12). The suppression of bile production and excretion under fasting conditions is most likely the reason for the observed increase (19). In order to exclude these obvious effects at the last sampling time of 24 h, the kinetic evaluation was limited to the range of 0–12 h. Table 2 summarizes the biokinetic parameters of predose-corrected 5-CH_3_-H_4_folate. The time to attain the maximum concentrations (*t*_max_) was highly variable among treatments. The results were surprising as the low-fat Camembert in the present study showed a much higher folate bioavailability (65–71%, c.f. Table 2) than the low-fat Camembert investigated in our previous study, when we found a mean bioavailability of 8.8% for all 24 volunteers (12). However, as both studies were conducted under virtually identical conditions and only a few weeks apart and the two volunteers of the present study also participated in the other one, a cross-study comparison [present pilot study vs. Ref. (12)] is possible. Both volunteers also revealed a tremendous lower folate bioavailability for the other Camembert as low as 9.2 and 16.4%, respectively, relative to the reference dose of the folic acid solution.

**Table 1:** **Folate distribution and sum of all folate vitamers in the test foods, mean ±SD (*n* = 3)**.

Food	Tetrahydrofolate (**μ**g/100 g)	5-Methyl-tetrahydrofolate (**μ**g/100 g)	5-Formyl-tetrahydrofolate (**μ**g/100 g)	10-Formylfolate (**μ**g/100 g)	Pteroylmonoglutamic acid (**μ**g/100 g)	Sum of folates (nmol/100 g)
Camembert cheese (present study)	17.7 ± 1.8	31.9 ± 4.5	38.6 ± 0.9	6.4 ± 0.6	n.d.	224 ± 18
Camembert cheese (12)	144.7 ± 12.9	46.2 ± 2.2	54.5 ± 4.1	40.2 ± 5.3	n.d.	647 ± 16

*n.d., not detectable*.

**Table 2:** **Summary table of baseline corrected 5-CH_3_-H_4_folate biokinetic parameter in plasma**.

Parameter^a^	Camembert		Oral solution	
*C*_max_ (nmol/L)	17.3 ± 12.8	(74)	5.11 ± 4.79	(94)
*t*_max_ (h)	24 (24, 24)		6 (4, 8)	
AUC_0–12_ (nmol⋅h/L)^b^	45.9 ± 5.96	(13)	65.0 (n.c.)	n.c.
Ratio estimate in % of reference baseline corrected and dose-normalized^c^	71/65			

*Doses of the testings were 448 and 453 nmol sum of folates *via* Camembert cheese and oral solution of pteroylmonoglutamic acid, respectively*.

*Tabulated values are arithmetic mean ± SD (CV%) of *n* = 2 subjects except for *t*_max_ where values are median (min, max); n.c., not calculated because AUC was not calculated in subject 2; reason: this subject had its last positive, pre–post-dose concentration difference at 4 h after dosing*.

*^a^*C*_max_ and *t*_max_ were determined within the 0- to 24-h interval*.

*^b^AUC_0–12_ is the *positive* AUC within the interval 0–12 h, i.e., concentrations falling below the individual predose values were discarded*.

*^c^Oral solution as reference, percentage based on the mean of AUC_0–12_/based on the subject with valid Camembert and oral solution AUC data*.

## Discussion

The reason for this may be explained by the different folate distributions in the two cheeses. The Camembert with the lower bioavailability (12) contained most of its folates (80%) in the rind, which is a more compact and firm matrix than the dough and may render the folates less accessible during gastrointestinal digestion. In contrast to this, in the present study, more than 60% of the folate vitamers could be found in the relatively soft dough matrix. Moreover, the major folate vitamer in the Camembert cheese with lower bioavailability was tetrahydrofolate (12), which is the least stable vitamer and therefore, particularly susceptible to degradation in the gastrointestinal environment (20).

The cross-study comparison [present pilot study vs. Ref. (12)] of the Camembert results of the two subjects, who participated in both studies, substantiated the different bioavailabilities on an intraindividual level (65–71 vs. 9–16%), despite the few volunteers in the present investigation. Moreover, as both studies were run under virtually identical conditions, they indicated the Camembert cheeses (different folate profiles and matrix distribution) as the determining factors. Also, the low number of recruited subjects in the present study is not unusual for pilot studies investigating the bioavailability and metabolism of bioactive food components (21, 22) with reported number(s) as low as one (23). However, we still want to emphasize that our study is an exploratory and limited one as there is only for one volunteer a complete data set.

Nevertheless, our results underline the dependence of folate bioavailability on the specific food product/type ingested. The reasons for the differences of folate bioavailability are still unclear. Previously, we assumed that (a) different kinetics and bioavailabilities of the folate vitamers (24) and particularly of the polyglutamate forms, (b) presence of deconjugase inhibitors, and (c) entrapping of folates in the food matrix may account for these differences. Therefore, the general folate bioavailability from foods of 50% as the basis for the definition of folate equivalents must be seriously questioned, and its generality requires further investigation. Possible approaches are further testing of foods in human studies with less expensive designs, such as sampling of dried blood spots (25) or the application of *in vitro* digestion models, which can be used as a cost- and time-efficient high throughput “screening tool.” The respective investigations are currently in progress.

## Conclusion

Although on a pilot scale, the results of the present study clearly demonstrate that a food product (e.g., Camembert cheese) cannot be regarded as a homogenous dietary “standard” with a predictable folate bioavailability. Mandatory folic acid fortification as successfully implemented in many countries, including the USA and Canada (6), is therefore a very efficient strategy to overcome these shortcomings in folate bioavailability and subsequently bioactivity.

## Author Contributions

MN, GN, and UO carried out the human study. SM developed the folate assay and analyzed the blood samples. TF performed the biokinetic calculations. The study was designed by MN, GN, and MR. The manuscript was written by MR, MN, GN, TF, and SM.

## Conflict of Interest Statement

The authors declare that the research was conducted in the absence of any commercial or financial relationships that could be construed as a potential conflict of interest.
